# A panel of free fatty acid ratios to predict the development of metabolic abnormalities in healthy obese individuals

**DOI:** 10.1038/srep28418

**Published:** 2016-06-27

**Authors:** Linjing Zhao, Yan Ni, Xiaojing Ma, Aihua Zhao, Yuqian Bao, Jiajian Liu, Tianlu Chen, Guoxiang Xie, Jun Panee, Mingming Su, Herbert Yu, Congrong Wang, Cheng Hu, Weiping Jia, Wei Jia

**Affiliations:** 1 Shanghai Key Laboratory of Diabetes Mellitus and Center for Translational Medicine, Shanghai Jiao Tong University Affiliated Sixth People’s Hospital, Shanghai 200233, China; 2 University of Hawaii Cancer Center, Honolulu, Hawaii 96813, United States; 3 Department of Endocrinology and Metabolism, Shanghai Jiao Tong University Affiliated Sixth People’s Hospital, Shanghai Diabetes Institute, Shanghai Clinical Center of Diabetes, Shanghai Key Laboratory of Diabetes Mellitus, Shanghai Key Clinical Center for Metabolic Disease, Shanghai 200233, China; 4 Department of Cell and Molecular Biology, John A. Burns School of Medicine, University of Hawaii at Manoa, Hawaii 96813, United States

## Abstract

Increasing evidences support that metabolically healthy obese (MHO) is a transient state. However, little is known about the early markers associated with the development of metabolic abnormalities in MHO individuals. Serum free fatty acids (FFAs) profile is highlighted in its association with obesity-related insulin resistance, type 2 diabetes mellitus (T2DM) and cardiovascular diseases (CVD). To examine the association of endogenous fatty acid metabolism with future development of metabolic abnormalities in MHO individuals, we retrospectively analyzed 24 [product FFA]/[precursor FFA] ratios in fasting sera and clinical data from 481 individuals who participated in three independent studies, including 131 metabolic healthy subjects who completed the 10-year longitudinal Shanghai Diabetes Study (SHDS), 312 subjects cross-sectionally sampled from the Shanghai Obesity Study (SHOS), and 38 subjects who completed an 8-week very low carbohydrate diet (VLCD) intervention study. Results showed that higher baseline level of oleic acid/stearic acid (OA/SA), and lower levels of stearic acid/palmitic acid (SA/PA) and arachidonic acid/dihomo-γ-linolenic acid (AA/DGLA) ratios were associated with higher rate of MHO to MUO conversion in the longitudinal SHDS. Further, the finding was validated in the cross-sectional and interventional studies. This panel of FFA ratios could be used for identification and early intervention of at-risk obese individuals.

Obesity, a global epidemic health problem, is associated with high risks of metabolic syndrome (MetS), type 2 diabetes mellitus (T2DM), and cardiovascular diseases (CVD)^1^. However, up to 30% of obese people are metabolically healthy^2^. These metabolically healthy obese (MHO) individuals have normal levels of circulating lipids, hormones, and inflammatory cytokines, their insulin sensitivity, hepatic enzyme function, and immune function are also normal, and they have no hypertension^3^. However, the metabolic normality in some of the MHO individuals may be short-lived. Although MHO people may not be at increased short-term risk of metabolic abnormalities^4^,^5^,^6^,^7^, studies with longer follow-up have found increased likelihood of T2DM, CVD and mortality in the MHO compared with metabolically healthy, normal-weight (MH-NW) subjects^8^,^9^,^10^. To date, factors that are associated with healthy and unhealthy obesity phenotypes remain unclear. Identification of early metabolic markers associated with the risk of developing metabolic disorders in MHO people will help us distinguish at-risk obese individuals, and delay or prevent the onset of metabolic diseases with proper interventions.

Free fatty acids (FFAs) are released from adipocytes into the circulation through lipolysis, and the concentration of circulating FFAs is usually elevated in obese subjects due to increased amount of adipose tissue. High levels of FFAs in the circulation are implicated in the pathogenesis of obesity-related insulin resistance, T2DM, and CVD^11^, possibly through increased generation of deleterious lipid metabolites^12^, proinflammatory cytokines^13^ and inducing oxidative stress and endoplasmic reticulum stress^14^,^15^. We have recently shown that several circulating unsaturated FFAs were predictive of future metabolic abnormalities in MHO individuals^16^. Compared with concentrations of individual FFAs, the ratios of [product FFA/[precursor FFA] may better reflect the status of endogenous fatty acid metabolism. In this study, we measured 24 [product FFA]/[precursor FFA] ratios, and assessed the associations between these ratios and the metabolic status of obese individuals, in order to uncover novel biomarkers which could be used to identify MHO individuals who are at-risk for metabolic disorders. This study examined samples collected from participants in 3 independent studies, including a 10-year longitudinal cohort of 131 metabolically healthy individuals from the Shanghai Diabetes Study (SHDS)^17^, a cross-sectional cohort of 312 subjects from the Shanghai Obesity Study (SHOS)^18^, and a controlled dietary intervention study of 38 obese subjects who completed 8-week of very low carbohydrate diet (VLCD)^19^.

## Results

### FFA ratios as predictive markers for the development of metabolic abnormalities in MHO

The subjects selected from the SHDS consisted of 69 MH-NW and 62 MHO at baseline, with clinical data and sera collected at both baseline and 10-year follow-up. The data were used in the present study to investigate whether MHO had higher risk of developing metabolic abnormalities than MH-NW, and to explore the potential role of FFA ratios as early metabolic markers to predict the development of metabolic abnormalities in MHO. At 10-year follow-up, 33 of 69 baseline MH-NW remained metabolically healthy (stable MH-NW) and other 36 became MU-NW (progressors to MU-NW). By contrast, 12 of 62 baseline MHO remained their metabolic health after 10 years (stable MHO), but 50 baseline MHO developed metabolic abnormalities at the 10-year follow-up (progressors to MUO). The incidence rate of metabolic abnormalities was 1.54-fold higher in MHO than in MH-NW during the 10 years.

The baseline clinical characteristics of MH-NW and MHO groups and four subgroups categorized by follow-up metabolic status were listed in Table 1. The participants in baseline MH-NW group were younger than the MHO. SBP, DBP, 2hPG, fasting insulin (FINS), 2 h INS, TG and HOMA-IR were significantly higher and Matsuda ISI was lower in the MHO group compared with NH-NW. Further, stable MHO had higher levels of 2hPG and TC, and lower level of insulin sensitivity relative to the stable MH-NW. However, all of baseline clinical parameters in Table 1 were comparable between the progressors to MU-NW and progressors to MUO. Only TG was significantly higher in both progressors to MUO and progressors to MU-NW compared with their stable MH subjects, respectively (*P* < 0.05).

**Figure 1:**
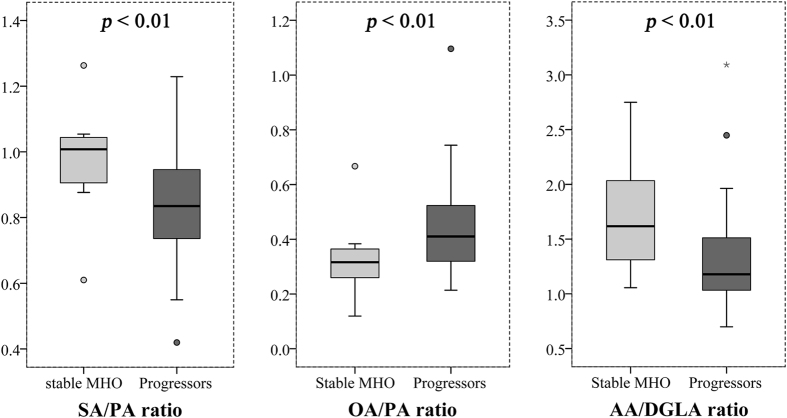


**Figure 2:**
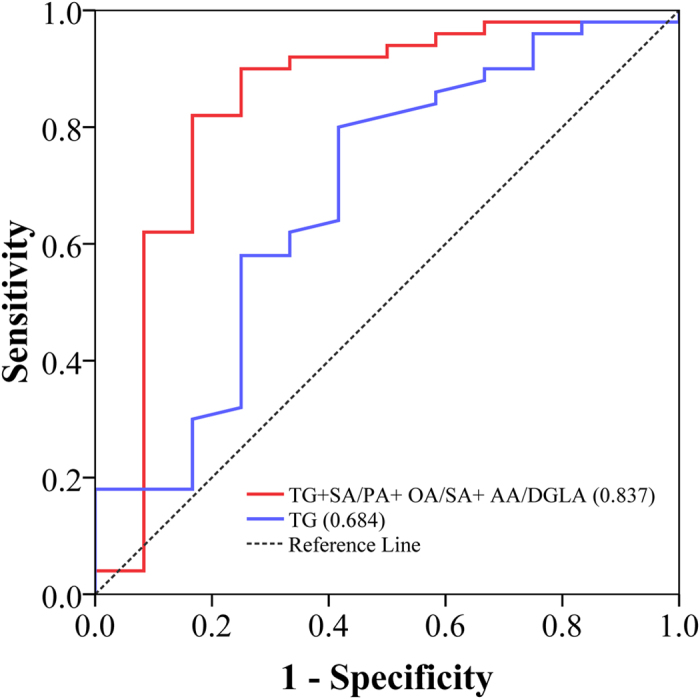
Relative contribution of a panel of FFAs ratios to predict metabolic abnormalities after 10-year in MHO individuals. Presented are ROC AUC comparing TG itself and the combination of TG with SA/PA, OA/SA and AA/DGLA. The combination model of three ratios and TG is computed by 1.748 × TG + 5.447 × OA/SA −0.601 × SA/PA −1.477 × AA/DGLA.

Among 24 [product FFA]/[precursor FFA] ratios, 3 were significantly altered (*P* < 0.01) in progressors to MUO compared with stable MHO, and these 3 ratios were SA/PA (C18:0/C16:0), OA/SA (C18:1 n9/C18:0), and AA/DGLA (C20:4 n6/C20:3 n6) (Fig. 1 and Supplementary Table S1). The predictive powers of the 3 ratios for the risk of developing metabolic abnormalities among the MHO were evaluated using multivariate logistic regression analysis (Table 2). The results showed that baseline level of OA/SA ratio was a positive predictor (adjusted odds ratios 2.38 [95% CI 1.05-5.40]), and SA/PA, AA/DGLA were negative predictors (0.59 [95% CI 0.36-0.98] and 0.85 [95% CI 0.72-0.99], respectively), independent of sex, age, BMI and TG. We also performed receiver operating characteristic (ROC) analysis on the 3 FFAs ratios and conventional predictor of TG, and found that the predictive value of 3 FFA ratios were better than it, with an area under the curve (AUC) of 0.755~0.767 compared to the AUC of 0.684 for TG. When the SA/PA, OA/SA, AA/DGLA ratios were combined with TG, the potential to distinguish the progressors to MUO and stable MHO was 0.837 as measured by ROC AUC (Fig. 2). By contrast, none of FFA ratios had significant difference between stable MH-NW and progressors to MU-NW.

### Associations of SA/PA, OA/SA and AA/DGLA with metabolic health and BMI

To further confirm the associations of SA/PA, OA/SA, AA/DGLA ratios with metabolic and BMI, we compared the 3 FFA ratios among the MH-NW, MHO and MUO groups in the 312 subjects selected from the SHOS. Table 3 shows the clinical characteristics of the three study groups. The MHO and MUO subjects had similar BMIs which were significantly higher than that of the MH-NW. Subjects in the MUO group were ~10 years older than those in the other two groups. As expected, FPG, 2hPG and HbA1c concentrations were all significantly higher in the MUO subjects compared with the other two groups. FINS, TG, LDL-C and HOMA-IR were significantly higher and HDL-C was lower in the MHO and MUO subjects compared with NH-NW, and these parameters were significantly different between the MHO and the MUO.

The 24 [product FFA]/[precursor FFA] ratios are summarized in Supplementary Table S2. Strikingly, the SA/PA, OA/SA and AA/DGLA ratios were all significantly altered in the MUO (*P* < 0.01) compared with both MH-NW and MHO groups (Table 3). Correlation analysis showed OA/SA was positively correlated with FPG, 2 h PG, HbA1c, TG, LDL-c, HOMA-IR, and negatively correlated with Matsuda ISI, while SA/PA and AA/DGLA were both positively correlated with Matsuda ISI, and negatively correlated with FPG, 2 h PG, HbA1c, TG, LDL-c, HOMA-IR (|r| = 0.15 ~ 0.59, *P* < 0.05) (Supplementary Table S3).

### Effects of diet-induced weight loss on SA/PA, OA/SA, and AA/DGLA ratios

SA/PA, OA/SA, and AA/DGLA ratios were analyzed for the 38 obese subjects from the dietary intervention study to examine the effect of weight loss on these FFA ratios. The clinical characteristics of the participants are summarized in Table 4. Eight weeks of VLCD resulted in a significant decrease of BMI) (3.1 kg/m^2^), significant reduction of subcutaneous and visceral fat areas, and improvement of insulin sensitivity. Compared with the baseline levels, OA/SA was significantly decreased while SA/PA and AA/DGLA increased after 8 weeks of VLCD intervention (*P* < 0.01 or 0.05) (Table 4). Correlation analysis showed the changes of OA/SA and AA/DGLA were negatively (r = −0.35, p = 0.03) and positively (r = 0.52, p < 0.01) correlated with the change of Matsuda ISI, respectively.

## Discussion

The present study highlighted the predictive value of SA/PA, OA/SA and AA/DGLA ratios for the development of metabolic abnormalities in MHO, i.e., MHO with higher OA/SA ratio had greater chance of being metabolically unhealthy within a 10-year period, while MHO with higher SA/PA and AA/DGLA ratios had lower chance of developing metabolic abnormalities. The predictive value of these 3 ratios is independent of age, sex, BMI, TG and with higher power (added 0.153 units of ROC area to the TG alone). The finding may contribute to more precise evaluation of the risk of metabolic disease in MHO, and enable early intervention to protect the vulnerable population. Prospective studies with long-term follow-ups have shown that MHO individuals had increased risk for developing metabolic abnormalities. For example, 37.1% of baseline MHO subjects became MUO, whereas 15.9% MH-NW were reclassified as MU-NW at 6-year follow-up in Pizarra study^20^. Similarly, in the North West Adelaide Health study, compared with metabolically healthy normal-weight subjects, the MHO were more likely to develop metabolic risk (15.5% vs. 33.1%) during 5.5–10.3 years of follow-up^4^. Other studies also reported that 42.1% metabolically healthy abdominal obese subjects developed MetS^9^ and 64.7% MHO became MUO at 10-year follow-up^21^. Furthermore, a systematic review evaluating the associations of metabolic health and BMI with CVD events reported that MHO individuals are at increased risk for adverse long-term CVD outcomes compared with MH-NW (RR = 1.24; 95% CI, 1.02 to 1.55), even in the absence of metabolic abnormalities^22^. In our longitudinal SHDS at 10-year follow-up, we found a higher incidence rate of metabolic abnormalities in MHO (80.6%, n = 62) compared with their MH-NW counterpart (52.2%, n = 69), which further proves that MHO is at higher risk of developing metabolic abnormalities. Factors that benefit metabolic health in obese people are yet to be fully understood. Previous cross-sectional analyses suggested MHO subject were more likely to be younger and female, with higher physical activity and fitness^23^,^24^. In addition, greater insulin sensitivity, smaller visceral and ectopic fat accumulation, higher adiponectin level and decreased circulating concentration of high-sensitive C-reactive protein (hsCRP), a surrogate marker of systemic inflammation, were suggested to be the protective factors in MHO phenotype^21^,^25^,^26^. In the analysis of samples collected from the SHDS longitudinal study, we found that, baseline TG was higher in the stable MHO group than the progressors to MUO (p = 0.049), but other baseline markers such as sex, age, plasma glucose and insulin, LDL, HDL and insulin sensitivity were comparable between the two groups. Notably different studies used different criteria to identify MHO. Here we defined MHO as the absence of components of metabolic syndrome by CDS, which was in line with some analyses^22^, but different from most studies in which allowed MHO to have one^4^,^27^, or even two^5^,^21^ metabolic syndrome components.

Chronic exposure to elevated concentration of FFAs promotes insulin resistance^28^ and impairs compensatory β-cell response^29^, which increased the subsequent risks of MetS, T2DM, and CVD. Previous studies confirmed that MHO had lower visceral, liver, and muscle fat contents than insulin-resistant obese people did, suggesting that MHO have a better abilities to trap FFAs in the adipose tissue^21^,^30^. Consistently, a cross-sectional study by Succurro *et al*.^31^ also observed that MUO subjects have increased levels of plasma total FFA compared with the MHO.

The profile of circulating FFAs is considered as a surrogate marker of fatty acid composition in the adipose tissue^32^. However, to our best knowledge, few studies have used individual FFA or the ratios of [product FFA]/[precursor FFA] to predict the incidence of metabolic abnormalities in MHO subjects. Challenge may be mainly from the development of easy, fast and reliable method to quantitate individual FFAs in the blood^11^ which only contain a fairly small fraction of FFAs among the total fatty acids in the circulation (0.2–0.7 mM FFAs vs. 10 mM total fatty acids)^33^. Our previous work developed a targeted metabolomics approach and quantitatively determined 40 serum FFAs to assess their associations with metabolic health in obese individuals, and we found that several unsaturated FFAs were associated with the risk of MetS and T2DM^16^. Because circulating FFA composition to some extent reflects the function of elongase and desaturase during endogenous fatty acid metabolism, we hypothesized that some key [product FFA]/[precursor FFA] ratios might be better metabolic markers for adverse outcomes of obesity. We found that SA/PA, OA/SA, and AA/DGLA ratios, which reflect stearoyl-CoA desaturase 1 (SCD-1), delta 5 desaturase (D5D) and *ELOVL* 6 activities, respectively, independently associated with the incidence of metabolic abnormalities in baseline MHO at 10-year follow-up. This novel finding was corroborated by a cross-sectional sampling from the SHOS, and a dietary intervention study. Our findings highlighted the importance of fatty acid metabolism in the maintenance of metabolic health in overweight/obese subject, and warrant future studies to investigate the roles of SCD-1, D5D, and ELOVL6 in adipocyte, which may affect FFA metabolism, transportation, storage and utilization.

The effects of some confounding factors such as gender and age on the changes of three key FFAs ratios were examined in this study. We performed gender-based subgroups analyses on MH-NW, MHO and MUO participants in the cross-sectional study, respectively. Results showed that at similar BMI and metabolic status, the levels of the OA/SA, SA/PA and AA/DGLA ratios in male and female participants had not statistically significant difference (p > 0.05). Similarly, none of them had the significant correlation with age in both SHDS and SHOS studies. Considering that the participants in baseline MH-NW group were younger (age range 21~39) than the MHO in the SHDS, we further investigate whether MHO had a higher risk of developing metabolic abnormalities than MH-NW under age-matched condition. Among 29 subjects less than 40 years old selected from 62 baseline MHO subjects, 23 of them (79.3%) developed metabolic abnormalities at 10-year follow-up, similar to the present results ignoring the age in that 50 of 62 baseline MHO (80.6%) progressed to MUO. These results confirmed that the confounding factors of gender and age did not affect our present findings.

There are several limitations in this study. Firstly, the subjects in the longitudinal SHDS were randomly recruited from two urban communities of Shanghai, China, but only ~20% (1250 of 5994) completed 10-year follow-up visit, which may result in selection bias in the cohort used in the present study. Secondly, the size of the cohort selected from the SHDS is moderate, and therefore the predictive value of three ratios should be validated larger cohorts, particularly in population-based prospective studies. Thirdly, the FFAs ratios at 10-year follow up were not measured. Fourthly, information on lifestyle and diet were not collected, which may result in covariate imbalance. Fifthly, a recent study reported that insulin sensitivity improved by about 26% in MUO but decreased by about 13% in MHO after a 6-month energy-restricted diet^34^, and it would be interesting to assess the differences of these key FFA ratios between MHO and MUO individuals after achieving similar diet-induced weight loss. Lastly, due to the potential effect of dietary habit on the fatty acids composition in adipose tissue, whether the findings in this study could translate across into other populations needs further validation. The main strength of this study is that as a retrospective study, we utilized clinical information and serum samples collected in 3 independent studies. Results from the longitudinal SHDS established potential cause-and-effect relationship between the FFA ratios and the incidence of metabolic abnormalities in the MHO, the cross-sectional sampling from the SHOS corroborated the association between the FFA ratios and metabolic abnormalities, and the dietary intervention study demonstrated that diet and BMI were influential factors on the FFA ratios.

In conclusion, we showed that MHO individuals had a higher risk of developing metabolic abnormalities over time compared with MH-NW in a communities-based Chinese population. The SA/PA, OA/SA and AA/DGLA ratios were associated with the incidence of metabolic abnormalities in MHO subjects, and had relatively high predictive value to identify overweight/obese individuals who are at-risk for metabolic abnormalities. Further study is needed to elucidate whether desaturase and elongase, such as SCD-1, D5D, and ELOVL6, play a causal role in metabolic dysregulation and could be novel therapeutic targets for prevention and treatment of metabolic deterioration in obese population.

## Methods

### Study design and participants

This retrospective study utilized clinical data and samples collected from participants enrolled in the following studies:

1. SHDS^17^: a 10-year community-based epidemiological survey initiated in 1998–2001, designed to assess the prevalence of diabetes and other metabolic disorders. Totally 5,994 individuals older than 15 years were enrolled from Shanghai, China, and 1,250 subjects completed the follow-up examination between 2010 and 2011. A total of 10.5% (131 out of 1250) subjects (mean age 37 years, 72.5% women) from SHDS met our inclusion criteria and were selected for the present study. The inclusion criteria included: 1) metabolically healthy at baseline; 2) no significant changes of body mass index (BMI) level between the baseline and follow-up (significant changes include NW → OW/OB, or OW/OB → NW). Among the 131 participants, 69 had normal weight, and other 62 were overweight or obese.
2. SHOS^18^: a prospective study designed to investigate the occurrence and development of metabolic syndrome and related disease, started in 2009 and enrolled 5000 participants older than 20 years from four communities in Shanghai, China. The present study included clinical data and samples from 312 adult participants in SHOS (mean age 49 years, 60.6% women). The criteria for selecting participants for the present study were as follow: 1) Those metabolically healthy participants with normal BMI (MH-NW, n = 132);2) Those metabolically healthy participants with overweight or obese state (MHO, n = 107); 3) Those metabolically unhealthy participants with overweight or obese state (MUO, n = 73). Any participants who had received antihypertensive therapy, lipid-lowering therapy, or a combination of antihypertensive and lipid-lowering therapy were excluded.
3. A controlled dietary intervention study^19^: an 8-week VLCD intervention study on 53 obese volunteers. The present study involved 38 subjects (mean age 33 years, 39.5% women, mean BMI 32.9 kg/m^2^) who completed the 8-week VLCD and their serum samples collected at baseline and eight weeks after intervention.

The protocol for this metabolomics study using samples from the three aforementioned studies was approved by the ethics committee at the Shanghai Jiao Tong University Affiliated Sixth People’s Hospital. Written informed consent was obtained from all participants.

### Definitions of overweight, obesity, and metabolic health

According to the Working Group on Obesity in China, for Chinese adults, obesity, overweight, and normal weight are defined as BMI ≥28 kg/m^2^, 28 kg/m^2^ >BMI ≥24 kg/m^2^, and BMI <24 kg/m^2^, respectively^35^. Being metabolically healthy means complete absence of any metabolic abnormalities that meet the Chinese Diabetes Society (CDS), metabolic syndrome diagnostic criteria^36^, including 1) systolic blood pressure (SBP) >140 mmHg or diastolic blood pressure (DBP) >90 mmHg; 2) fasting plasma triglyceride (TG) >1.7 mmol/L; 3) fasting plasma glucose (FPG) >6.1 mmol/L or 2-hour after meal plasma glucose (2hPG) >7.8 mmol/L; 4) High-density lipoprotein (HDL) ≥0.9 mmol/L in male or ≥1.0 mmol/L in female.

Based on the changes of metabolic parameters between baseline and follow-up, participants in the SHDS longitudinal study were separated into four groups: 1) **stable MH-NW**: BMI <24 kg/m^2^ and no metabolic abnormalities at baseline and follow-up; 2) **progressors to MU-NW**: BMI <24 kg/m^2^ no metabolic abnormalities at baseline, remained normal weight but became metabolically unhealthy at follow-up; 3) **stable MHO**, BMI ≥24 kg/m^2^ and no metabolic abnormalities at baseline and follow-up; 4) **progressors to MUO**: BMI ≥24 kg/m^2^ and no metabolic abnormalities at baseline, remained being overweight or obese and became metabolically unhealthy at follow-up. Participants selected from the SHOS were divided into three group: 1) **MH-NW:** BMI <24 kg/m^2^ and no metabolic abnormalities; 2) **MHO**: BMI ≥24 kg/m^2^ and no metabolic abnormalities; 3) **MUO**: BMI ≥24 kg/m^2^ and met at least two CD metabolic syndrome diagnostic criteria.

### Anthropometric indices and laboratory measurements

Each participant had a physical examination including measurement of height, weight, waist circumference and blood pressure. BMI was calculated as weight (kg) divided by squared height (m^2^). Waist circumference (WC) was measured at the horizontal plane between the inferior costal margin and the iliac crest on the mid-axillary line. Blood pressure was the average of three measurements made with a sphygmomanometer at two minute intervals.

After a fasting venous blood sample was collected, each participant received a 75 g oral glucose tolerance test. Plasma glucose levels were measured by the glucose oxidase method. Serum insulin was assayed using bio-antibody technique (Linco, St Louis, MO, USA). Serum lipid profiles including total cholesterol (TC), serum total triglyceride (TG), high-density lipoprotein cholesterol (HDL-C) and low-density lipoprotein cholesterol (LDL-C), were determined by standard commercial methods on a parallel, multichannel analyser (Hitachi 7600-020, Tokyo, Japan). An experienced technician measured glycated haemoglobin A1c (HbA1c) of de-identified samples using high performance liquid chromatography (HLC-73G7, Tosoh, Japan). Insulin resistance and insulin sensitivity were evaluated using the following formulas: (1) homeostasis model assessment of insulin resistance (HOMA-IR) = fasting insulin×fasting glucose/22.5; (2) Matsuda insulin sensitivity index (Matsuda ISI) = 10,000/√ (fasting insulin×fasting glucose×mean insulin during OGTT×mean glucose during OGTT)^37^.

Subcutaneous and visceral fat areas were assessed by Philips Achieva 3.0T MRI system (Philips Medical Systems, Eindhoven, The Netherlands) using standard array coils with the subject in a supine position as previously reported^19^.

### Sample preparation and FFAs analysis

All of serum samples were stored at −80 °C until use. Sample preparation were performed according to a modified protocol based on Püttmann *et al*.^33^. Forty FFAs were analyzed using an ACQUITY ultra-performance liquid chromatography (UPLC) system (Waters Corporation, Milford, USA) equipped with a binary solvent delivery system and an auto-sampler (Waters Corporation, Milford, USA), coupled to a tandem quadrupole-time-of-flight (Q-TOF) mass spectrometry (Waters Corporation, Milford, USA)^16^. For details, see the Supplementary information. A mixture of all the reference standards at an appropriate concentration was prepared and run after every ten serum samples for quality control. Twenty-four FFAs ratios were calculated by the absolute concentrations of product- to-precursor.

### Statistical analysis

The raw data produced by UPLC-QTOFMS were initially processed using TargetLynx applications manager version 4.1 (Waters Corp., Milford, MA) to detect peak signals, obtain calibration equations, and calculate the absolute concentration of each FFA^16^. Manual examination and correction were needed to ensure data quality.

Data are presented as mean ± SD or median ± SE. Group values were compared using the Mann-Whitney test for continuous variables or χ^2^ for nominal variables. Simple associations were tested by spearman correlation coefficients (r) between calculated FFAs ratios and clinical measures. Logistic regression was used to predict metabolic abnormalities risk; odds ratios and 95% CIs were presented. Performance of different logistic models was assessed as the receiver operating characteristic area under the curve (ROC AUC). Statistical significance was defined as two-tailed *P* < 0.05. All statistical computing and graphics were carried out using SPSS 16.0 (SPSS, Chicago, IL, USA).

## Additional Information

**How to cite this article**: Zhao, L. *et al*. A panel of free fatty acid ratios to predict the development of metabolic abnormalities in healthy obese individuals. *Sci. Rep.* **6**, 28418; doi: 10.1038/srep28418 (2016).

## Supplementary Material

Supplementary Information

## Figures and Tables

**Table 1:** 10-year longitudinal study: baseline clinical characteristics of MH-NW and MHO groups and four subgroups categorized by follow-up metabolic outcomes.

	Baseline MH-NW			Baseline MHO		
	Total (n = 69)	Stable MH-NW (n = 33)	Progressors to MU-NW (n = 36)	Total (n = 62)	Stable MHO (n = 12)	Progressors to MUO (n = 50)
Women (%)	71	73	69	74	92	70
Age (years)^¶¶^	32 ± 6.7	30 ± 6.8	33.8 ± 6.2*	43 ± 13	40 ± 13^†^	44 ± 13
BMI (kg/m^2^)^¶¶^	21.4 ± 1.4	21.4 ± 1.4	21.4 ± 1.4	26.9 ± 2.5	26.9 ± 1.6^‡^	26.9 ± 2.6
SBP (mmHg)^¶^	109 ± 11	109 ± 11	108 ± 10	114 ± 12	109 ± 13	116 ± 12
DBP (mmHg)^¶¶^	71 ± 6	71 ± 6	72 ± 7	74 ± 6	72 ± 9	74 ± 5
FPG (mmol/L)	4.6 ± 0.4	4.6 ± 0.4	4.7 ± 0.5	4.7 ± 0.4	4.9 ± 0.4	4.7 ± 0.4
2 hPG (mmol/L)^¶¶^	4.6 ± 1.0	4.4 ± 0.9	4.8 ± 1.1	5.2 ± 1.0	5.1 ± 0.7^†^	5.3 ± 1.1
FINS (μU/mL)^¶^	5.8 ± 2.6	5.7 ± 2.5	5.9 ± 2.8	7.2 ± 3.3	7.4 ± 3.0	7.1 ± 3.4
2 h INS (μU/mL)^¶¶^	27.7 ± 16.5	26.9 ± 16.2	28.5 ± 17	40.2 ± 27.9	43.6 ± 39.6	39.4 ± 24.8
TC (mmol/L)	4.0 ± 0.5	3.9 ± 0.4	4.2 ± 0.4**	4.1 ± 0.5	4.2 ± 0.5^†^	4.1 ± 0.5
TG (mmol/L)^¶¶^	0.9 ± 0.3	0.8 ± 0.3	1 ± 0.3*	1.1 ± 0.3	0.9 ± 0.3	1.1 ± 0.3*
HDL-c (mmol/L)	1.4 ± 0.2	1.3 ± 0.2	1.4 ± 0.2	1.3 ± 0.2	1.3 ± 0.2	1.3 ± 0.2
LDL-c (mmol/L)	2.6 ± 0.5	2.5 ± 0.5	2.7 ± 0.6	2.7 ± 0.4	2.7 ± 0.4	2.7 ± 0.4
HOMA-IR^¶¶^	1.2 ± 0.6	1.2 ± 0.6	1.2 ± 0.7	1.5 ± 0.7	1.6 ± 0.7^†^	1.5 ± 0.7
Matsuda ISI^¶¶^	292 ± 191	301 ± 222	278 ± 159	208 ± 114	212 ± 160^†^	207 ± 102

Entries are mean ± SD. *P* values were calculated by means of Mann-Whitney test for continuous variables and χ^2^ test for categorical variables, respectively. ^¶^*P* < 0.05, ^¶¶^*P* < 0.01 for the difference between baseline MH-NW and MHO. *P < 0.05; **P < 0.01 for the difference between stable MH-NW and progressors to MU-NW, or between stable MHO and progressors to MUO. ^†^*P* < 0.05, ^‡^*P* < 0.01 for the difference between stable MHO and stable MH-NW.

**Table 2:** 10-year longitudinal study: multivariate logistic regression analysis for the association of baseline SA/PA, OA/SA and AA/DGLA with metabolic abnormalities after 10 years in the MHO phenotype.

Variables	OR (95% CI)^1^	*P*^1^	OR (95% CI)^2^	*P*^2^
SA/PA (C18:0/C16:0)	0.57 (0.35–0.92)	**0.021**	0.59 (0.36–0.98)	**0.041**
OA/SA (C18:1n9/C18:0)	2.70 (1.20–6.08)	**0.017**	2.38 (1.05–5.40)	**0.038**
AA/DGLA (C20:4n6/C20:3n6)	0.85 (0.73–0.99)	**0.033**	0.85 (0.72–0.99)	**0.036**

Subjects with metabolic abnormalities at follow-up (n = 50) were compared with those MHO at the baseline and follow-up examinations (n = 12). ORs and their 95% CIs were obtained from logistic regression analyses. Bold type indicates statistical significance (*P* < 0.05).

^1^Adjusted for sex, age, BMI.

^2^Adjusted for sex, age, BMI and TG.

**Table 3:** Cross sectional study: clinical characteristics of MH-NW, MHO and MUO subjects from SHOS.

	MH-NW (n = 132)	MHO (n = 107)	MUO (n = 73)
Women (%)	64	64	49
Age (years)	46 ± 10	46 ± 8	57 ± 7^‡^
BMI (kg/m^2^)	20.5 ± 0.7	27.1 ± 1.9**	27.7 ± 2.6
WC (cm)	73 ± 5	88 ± 8**	92 ± 7§^‡^
SBP (mmHg)	115 ± 11	116 ± 11	138 ± 22^‡^
DBP (mmHg)	72 ± 8	75 ± 8**	82 ± 14^‡^
FPG (mmol/L)	5.0 ± 0.4	5.1 ± 0.4**	8.0 ± 2.6^‡^
2hPG (mmol/L)	5.6 ± 1.1	5.7 ± 1.0	14.9 ± 4.3^‡^
HbA1c (%)	5.4 ± 0.3	5.4 ± 0.4	7.1 ± 1.4§^‡^
FINS (μU/mL)	5.8 ± 2.6	9.1 ± 4.2	11.0 ± 5.2^‡^
2 h INS (μU/mL)	27.8 ± 16.8	36.8 ± 24.0	69.7 ± 50.4^‡^
TC (mmol/L)	4.3 ± 0.6	4.3 ± 0.5	5.4 ± 1.0^‡^
TG (mmol/L)	0.8 ± 0.3	1.0 ± 0.3**	2.4 ± 1.5^‡^
HDL-c (mmol/L)	1.6 ± 0.3	1.4 ± 0.3**	1.3 ± 0.2^‡^
LDL-c (mmol/L)	2.5 ± 0.5	2.7 ± 0.4**	3.3 ± 0.8^‡^
HOMA-IR	1.3 ± 0.6	2.1 ± 1.0**	3.9 ± 2.0^‡^
Matsuda ISI	177 ± 113	114 ± 52**	63 ± 26§^‡^
SA/PA (C18:0/C16:0)	0.99 ± 0.12	1.02 ± 0.10	0.85 ± 0.11^‡^
OA/SA (C18:1 n9/C18:0)	0.65 ± 0.31	0.56 ± 0.23*	0.85 ± 0.30^‡^
AA/DGLA (C20:4 n6/C20:3 n6)	8.61 ± 1.69	8.58 ± 1.89	7.56 ± 2.07^‡^

Entries are mean ± SD. *P* values were calculated by means of the Mann-Whitney test for continuous variables and χ^2^ test for categorical variables, respectively. *P < 0.05, **P < 0.01 for the difference between MHO and MH-NW; ^†^*P* < 0.05, ^‡^*P* < 0.01 between MHO and MUO.

**Table 4:** Dietary interventional study: clinical characteristics of obese subjects before and after 8-week VLCD (n = 38).

	Baseline	After 8 weeks
Women (%)	40	–
Age (years)	33 ± 9	–
BMI (kg/m^2^)	32.7 ± 4.6	29.8 ± 4.1**
WC (cm)	104.5 ± 12.23	99.03 ± 9.84*
SBP (mmHg)	135.5 ± 19.68	121.86 ± 12.5**
DBP (mmHg)	82.68 ± 11.77	74.08 ± 9.91**
FPG (mmol/L)	5.24 ± 0.91	5.19 ± 0.46
2 hPG (mmol/L)	7.5 ± 1.69	6.93 ± 1.72
FINS (μU/mL)	23.29 ± 29.61	12 ± 8.93**
2 h INS (μU/mL)	133.46 ± 72.69	72.92 ± 51.64**
TC (mmol/L)	5.19 ± 0.81	4.83 ± 0.91*
TG (mmol/L)	2.08 ± 1.7	1.1 ± 0.54**
HDL-c (mmol/L)	1.16 ± 0.31	1.18 ± 0.27
LDL-c (mmol/L)	3.09 ± 0.78	3.16 ± 0.8
HOMA-IR	6.39 ± 11.45	2.86 ± 2.37*
Matsuda ISI	67.66 ± 49.41	123.67 ± 78.39**
SFA (cm^2^)	348.11 ± 116.33	268.13 ± 99.93**
VFA (cm^2^)	117.11 ± 40.36	81.36 ± 21.91**
SA/PA (C18:0/C16:0)	0.92 ± 0.09	0.97 ± 0.11**
OA/SA (C18:1 n9/C18:0)	0.70 ± 0.26	0.62 ± 0.33*
AA/DGLA (C20:4 n6/C20:3 n6)	7.17 ± 1.62	8.43 ± 2.24*

Entries are mean ± SD. *P* values were calculated by means of Mann-Whitney test. *P < 0.05, **P < 0.01.
