# Supplementary material for: A panel of free fatty acid ratios to predict the development of metabolic abnormalities in healthy obese individuals
Source: Sci Rep. 2016 Jun 27;6:28418. doi: 10.1038/srep28418 (PMC4921829; doi:10.1038/srep28418)
Supplement: Supplementary Information [file srep28418-s1.pdf]

Supplementary information

A panel of free fatty acid ratios to predict the development of metabolic abnormalities in healthy obese individuals

Linjing Zhao [1,2], Xiaojing Ma[3], Aihua Zhao[1], Yuqian Bao[1,3], Jiajian Liu[1], Tianlu Chen [1], Guoxiang Xie [2], Jun Panee [4], Mingming Su [2], Herbert Yu [2], Congrong Wang [1], Cheng Hu [1], Weiping Jia [1,3*], and Wei Jia [1,2*]

[1] Shanghai Key Laboratory of Diabetes Mellitus and Center for Translational Medicine, Shanghai Jiao Tong University Affiliated Sixth People's Hospital, Shanghai 200233, China.

[2] University of Hawaii Cancer Center, Honolulu, Hawaii 96813, United States.

[3] Department of Endocrinology and Metabolism, Shanghai Jiao Tong University Affiliated Sixth People's Hospital, Shanghai Diabetes Institute, Shanghai Clinical Center of Diabetes, Shanghai Key Laboratory of Diabetes Mellitus, Shanghai Key Clinical Center for Metabolic Disease, Shanghai 200233, China.

[4] Department of Cell and Molecular Biology, John A. Burns School of Medicine, University of Hawaii at Manoa, Hawaii 96813, United States.

***Corresponding authors:**
Dr. Weiping Jia
Department of Endocrinology and Metabolism, Shanghai Jiao Tong University Affiliated Sixth People's Hospital, Shanghai Diabetes Institute, Shanghai Clinical Center of Diabetes, Shanghai Key Laboratory of Diabetes Mellitus, Shanghai Key Clinical Center for Metabolic Disease
600 Yishan Road, Shanghai 200233, China
Phone: +86-13818888939
Email: wpjia@sjtu.edu.cn

Dr. Wei Jia
Wei Jia, Ph.D., Professor
Director, Center for Translational Medicine, Shanghai Jiao Tong University Affiliated Sixth People's Hospital, 600 Yishan Road, Shanghai 200233, China.

Associate Director, University of Hawaii Cancer Center
701 Ilalo Street, Honolulu, HI 96813, USA.

Tel: (86)-21-24056374 (Shanghai); (808) 564-5823 (Hawaii)
Email: wjia@cc.hawaii.edu

Serum Sample preparation

In brief, 10 µL of isotope labeled internal standard (5 µg/mL of nonadecanoic-*d*37 acid) was added to each 40 µL of serum sample, followed by 500 µL of isopropyl / hexane/ phosphoric acid (2M) (40/10/1 by vol). Samples were then vortexed for 2 min. After incubating at room temperature for 20 min, 400 µL of n-hexane and 300 µL of water were added, vortexed and centrifuged at 12,000 rpm for 10 min. Then 400 µL of the upper organic layer was transferred to a new tube and 400 µL of n-hexane was added to the lower layer for further extraction. After vortex and centrifuge, all of the upper organic phase was then combined with the first supernatant and dried under vacuum. The residue was reconstituted with 80 µL of methanol and subjected to ultra-performance liquid chromatography quadrupole-time-of-flight mass spectrometry (UPLC-QTOFMS) analysis.

UPLC-QTOFMS analysis

An ACQUITY-UPLC system (Waters Corporation, Milford, USA) equipped with a binary solvent delivery system and an auto-sampler (Waters Corporation, Milford, USA) was employed for the separation on a 100 cm × 2.1 mm BEH C_{18} column with 1.7 µm particles at 40 ◦C (Waters Corporation, Milford, USA). The optimal mobile phase consisted of water (solvent A) and acetonitrile/isopropyl alcohol (80/20 by vol, solvent B) and the flow rate was set at 400 µL/min. The injection volume was of 5 µL. A gradient elution condition was applied as follows: 70% B over 0 ~ 2 min, 70% ~ 75% B over 2 ~ 5 min:, 75% ~ 80% B over 5 ~ 10 min:, 80% ~ 90% B over 10 ~ 13min:, 90% ~ 100% B over 13 ~ 16 min, maintained for 5 min, then returned to 70% B over 21 ~ 22.5 min and re-equilibrated for 1.5 min.

The mass spectrometric data was collected using a tandem quadrupole-time-of-flight (Q-TOF) mass spectrometry (Manchester, UK). ESI was used as the ionization source and the analysis was carried out in the negative mode. The following parameters were used: capillary voltage, 2.5 KV; sampling cone, 55 V; extraction cone, 4V; desolvation temperature, 450 ◦C; source temperature, 150 ◦C; desolvation gas flow, 650 L/h; and cone gas flow, 50 L/h.

Supplementary Table S1. **10-year longitudinal study: baseline serum product/precursor FFAs concentrations ratios in MH-NW or MHO subjects and four groups categorized by follow-up metabolic status**. Entries are median ± SE. *P* values were calculated by means of Mann-Whitney test. . ¶ *P*<0.05, ¶¶ *P*<0.01 for the difference between baseline MH-NW and MHO. * *P*<0.05, ** *P*<0.01 for the difference between stable MHO and progressors to MUO. † *P*<0.05 for the difference between stable MHO and stable MH-NW.

	**Baseline MH-NW**			**Baseline MHO**		
	Total (n=69)	Stable MH-NW (n=33)	Progressors to MU-NW(n=36)	Total (n=62)	Stable MHO (n=12)	Progressors to MUO (n=50)
C16:1 n7/16:0	0.029±0.001	0.029±0.002	0.030±0.002	0.032±0.001	0.030±0.003	0.032±0.002
C18:1 n9/C18:0	0.38±0.02	0.41±0.03	0.35±0.03	0.38±0.02	0.32±0.04 †	0.41±0.02**
C18:1 n9/C16:1 n7	11.33±0.47	11.58±0.81	10.82±0.51	10.86±0.32	11.14±0.67	10.76±0.37
C18:2 n6/C18:1 n9	2.08±0.09	2.16±0.14	2.07±0.12	2.06±0.10	2.06±0.21	2.07±0.11
C18:3 n3/C18:2 n6	0.062±0.003	0.060±0.004	0.064±0.004	0.064±0.002	0.065±0.005	0.064±0.002
C18:3 n6/C18:2 n6	0.012±0.001	0.011±0.001	0.012±0.001	0.013±0.001	0.016±0.002	0.013±0.001
C20:3 n6/C20:2 n6	12.74±0.83	13.21±1.28	12.74±1.09	13.52±0.71	10.64±1.33	13.64±0.81
C20:4 n6/C20:3 n6 ¶¶	1.50±0.07	1.64±0.14	1.45±0.06	1.26±0.06	1.62±0.15	1.18±0.06**
C22:5 n6/C22:4 n6	0.90±0.03	0.86±0.04	0.92±0.03	0.92±0.03	0.90±0.05	0.92±0.04
C22:6 n3/C22:5 n3 ¶	22.85±0.74	21.44±1.24	23.58±0.85	19.95±0.69	22.13±1.67	19.22±0.76
C14:0/C12:0	39.67±4.09	41.13±7.48	39.19±3.69	48.05±22.35	49.34±8.19	47.88±27.66
C16:0/C14:0	7.34±0.44	7.42±0.67	7.12±0.58	7.52±0.39	5.45±0.68 †	7.74±0.44*
C18:0/C16:0	0.90±0.03	0.88±0.04	0.92±0.04	0.86±0.02	1.01±0.04 †	0.84±0.02**
C20:0/C18:0	0.013±0.000	0.040±0.000	0.014±0.000	0.014±0.000	0.014±0.001	0.014±0.000
C22:0/C20:0	0.069±0.004	0.073±0.008	0.067±0.004	0.069±0.003	0.069±0.007	0.068±0.004
C24:0/C22:0	0.11±0.01	0.12±0.02	0.11±0.02	0.12±0.01	0.18±0.06 †	0.11±0.01*
C20:1 n9/C18:1 n9	0.028±0.002	0.029±0.003	0.027±0.002	0.025±0.001	0.030±0.003	0.025±0.001
C22:1 n9/C20:1 n9	0.51±0.05	0.51±0.09	0.51±0.07	0.52±0.05	0.63±0.13	0.51±0.05
C24:1 n9/C22:1 n9 ¶	0.047±0.004	0.047±0.007	0.048±0.005	0.057±0.005	0.050±0.009	0.058±0.006
C20:2 n6/C18:2 n6	0.016±0.001	0.016±0.001	0.017±0.001	0.017±0.000	0.017±0.001	0.017±0.000
C22:2 n6/C20:2 n6	0.032±0.003	0.032±0.005	0.034±0.003	0.033±0.001	0.039±0.003	0.033±0.001
C20:3 n6/C18:3 n6	8.89±0.80	9.88±1.42	8.82±0.80	8.47±4.75	7.68±1.11	8.61±5.88
C22:5 n3/C20:5 n3	0.59±0.04	0.60±0.05	0.59±0.05	0.58±0.04	0.53±0.16	0.59±0.04
C22:4 n6/C20: 4 n6	0.027±0.001	0.026±0.001	0.028±0.001	0.028±0.001	0.026±0.002	0.029±0.001

Supplementary Table S2. **Cross-sectional study: serum product/precursor FFAs concentrations ratios in three groups defined by metabolic and BMI status**. *$P<0.05$, ** $P<0.01$ for the difference between MHO and MH-NW by the Mann-Whitney test; † $P<0.05$, ‡ $P<0.01$ between MHO and MUO.

	**MH-NW**	**MHO**	**MUO**
16:1 n7/16:0	0.043±0.002	0.037±0.002*	0.050±0.003 ‡
C18:1 n9/C18:0	0.62±0.03	0.54±0.02*	0.88±0.04 ‡
C18:1 n9/C16:1 n7	14.33±0.32	14.60±0.35	14.28±0.40
C18:2 n6/C18:1 n9	1.42±0.04	1.51±0.04*	1.51±0.04
C18:3 n3/C18:2 n6	0.036±0.001	0.034±0.001	0.030±0.002
C18:3 n6/C18:2 n6	0.013±0.001	0.013±0.000	0.012±0.000
C20:3 n6/C20:2 n6	1.02±0.02	1.14±0.03**	1.22±0.03 †
C20:4 n6/C20:3 n6	8.70±0.15	8.42±0.18	7.27±0.24 ‡
C22:5 n6/C22:4 n6	0.58±0.01	0.59±0.01	0.51±0.01 ‡
C22:6 n3/C22:5 n3	6.76±0.31	8.07±0.40**	7.22±0.49
C14:0/C12:0	46.07±55.40	51.52±49.47	50.81±8.58
C16:0/C14:0	6.97±0.17	6.63±0.19	5.79±0.22
C18:0/C16:0	0.99±0.01	1.01±0.01	0.84±0.01 ‡
C20:0/C18:0	0.023±0.000	0.022±0.000	0.021±0.000 †
C22:0/C20:0	0.063±0.002	0.062±0.002	0.069±0.003
C24:0/C22:0	0.25±0.01	0.24±0.01	0.29±0.02 ‡
C20:1 n9/C18:1 n9	0.011±0.000	0.011±0.000	0.009±0.000 ‡
C22:1 n9/C20:1 n9	0.30±0.02	0.30±0.03	0.19±0.02 ‡
C24:1 n9/C22:1 n9	0.46±0.02	0.49±0.03	0.54±0.04
C20:2 n6/C18:2 n6	0.017±0.001	0.016±0.000	0.014±0.000‡
C22:2 n6/C20:2 n6	0.039±0.001	0.04±0.001	0.036±0.001 ‡
C20:3 n6/C18:3 n6	1.41±0.03	1.43±0.04	1.38±0.05
C22:5 n3/C20:5 n3	1.28±0.04	1.13±0.04*	1.51±0.06 ‡
C22:4 n6/C20:4 n6	0.12±0.002	0.11±0.002*	0.12±0.003

Supplementary Table S3. Cross-sectional study: spearman correlations of the SA/PA, OA/SA and AA/DGLA ratios with plasma glucose levels, lipid profile and insulin resistance/sensitivity indexes in both MHO and MUO. **P*<0.05; ** *P*<0.01.

	SA/PA	OA/SA	AA/DGLA
FPG	-0.48 **	0.36**	-0.17 *
2h PG	-0.59 **	0.47**	-0.25 **
HbA1c	-0.58 **	0.42**	-0.18 *
TC	-0.52 **	0.43**	-0.12
TG	-0.55 **	0.42**	-0.31 **
HDL-c	0.20 **	-0.16 *	0.08
LDL-c	-0.25 **	0.15*	-0.17 *
HOMA-IR	-0.30 **	0.15*	-0.26 **
Matsuda ISI	0.36 **	-0.23 **	0.25**

Supplementary Table S4. Dietary interventional study: serum product/precursor FFAs concentrations ratios before and after 8-week VLCD. *P<0.05; ** P<0.01 by the Mann-Whitney test.

	Baseline	After 8 weeks
C16:1 n7/16:0	0.05±0.004	0.04±0.004
C18:1 n9/C18:0	0.67±0.04	0.55±0.05*
C18:1 n9/C16:1 n7	12.27±0.58	12.18±0.96
C18:2 n6/C18:1 n9	1.35±0.06	1.33±0.05
C18:3 n3/C18:2 n6	0.031±0.002	0.028±0.001
C18:3 n6/C18:2 n6	0.013±0.001	0.011±0.001
C20:3 n6/C20:2 n6	1.14±0.05	1.05±0.04
C20:4 n6/C20:3 n6	7.20±0.26	8.02±0.36*
C22:5 n6/C22:4 n6	0.52±0.01	0.55±0.01
C22:6 n3/C22:5 n3	5.34±0.25	6.23±0.30*
C14:0/C12:0	18.32±1.87	24.22±9.62*
C16:0/C14:0	7.44±0.97	7.14±0.36
C18:0/C16:0	0.90±0.02	0.99±0.02**
C20:0/C18:0	0.023±0.001	0.027±0.001**
C22:0/C20:0	0.061±0.003	0.053±0.005*
C24:0/C22:0	0.24±0.017	0.26±0.021
C20:1 n9/C18:1 n9	0.009±0.001	0.010±0.001
C22:1 n9/C20:1 n9	0.25±0.04	0.33±0.03
C24:1 n9/C22:1 n9	0.49±0.04	0.39±0.04
C20:2 n6/C18:2 n6	0.014±0.001	0.015±0.001
C22:2 n6/C20:2 n6	0.040±0.001	0.039±0.001
C20:3 n6/C18:3 n6	1.34±0.062	1.31±0.052
C22:5 n3/C20:5 n3	1.44±0.072	1.11±0.071*
C22:4 n6/C20:4 n6	0.14±0.005	0.14±0.006
